# Adjusted Unit Value Transfer as a Tool for Raising Awareness on Ecosystem Services Provided by Constructed Wetlands for Water Pollution Control: An Italian Case Study

**DOI:** 10.3390/ijerph18041531

**Published:** 2021-02-05

**Authors:** Anacleto Rizzo, Giulio Conte, Fabio Masi

**Affiliations:** 1 IRIDRA SRL, Via La Marmora 51, 50121 Florence, Italy; fmasi@iridra.com; 2 Ambiente Italia SRL, Via Carlo Poerio 39, 20129 Milan, Italy; giulio.conte@ambienteitalia.it

**Keywords:** constructed wetland, treatment wetland, ecosystem service, value transfer, nature-based solution, green-blue infrastructure

## Abstract

Constructed wetlands (CWs) are nature-based solutions (NBS) for water pollution control that can also be designed to be multipurpose in terms of additional ecosystem services (ESs), such as biodiversity support and social benefits. Awareness about additional ESs of CWs can be raised with value transfer (VT) methods for ESs monetization, in particular, the simplified adjusted unit VT method. A multi-criteria analysis (MCA) was performed to compare grey and green infrastructure alternatives for the management of a combined sewer overflow in the Buccinasco town (Italy), in which the criteria related to ESs were monetized with an adjusted VT method (B£ST software). The results highlighted the potential interest in the implementation of the green infrastructure in a new urban park, due to the activation of additional ESs of interest, such as health and recreational aspects. The results were also confirmed by a sensitivity analysis, which simulated the variation of preferences among different stakeholder groups (e.g., citizens, environmentalists). In conclusion, this work provided a transparent methodology to support decisions regarding green and grey infrastructure, allowing to evaluate additional ESs from the beginning of the decision stage with low cost and efforts.

## 1. Introduction

Constructed wetlands (CWs, also known as treatment wetlands) are a well-known nature-based solution (NBS) for wastewater treatment and water pollution control. Recently, the CW potential to be a multipurpose NBS and green blue infrastructure is also gaining momentum [1,2]. CWs can contribute to flood mitigation in urban and semi-urban catchments [3], support biodiversity [4], produce biomass for both energy source [5] and CO_2_ sequestration [6,7]. Moreover, social benefits can be also provided in terms of development of new recreational sites [8].

Despite the advantages of the multiple ecosystem services (ESs) that can be activated, CWs face the same limitations as other NBSs, i.e., the need to properly quantify and display the provided benefits in order to inform decision makers [9]. To this aim, ESs evaluation can help deliver the advantages of CWs; a significant example is the Gorla Maggiore Water Park, a CW for combined sewer overflow (CSO) treatment. Designed as a multipurpose NBS, excellent water quality performance was reported in Masi et al. [10] and ESs were evaluated in the work of Liquete et al. [11], who highlighted significantly better performance in terms of biodiversity support and social benefits of green infrastructures in comparison to grey ones. Moreover, Reynaud et al. [12] registered a higher willingness to pay for green infrastructure compared to grey infrastructure on the same site, even greater if the green infrastructure was included in a park area. The success of the Gorla Maggiore Water Park convinced a key decision maker, the regional Public administrator (Lombardia Region), to spur the implementation of NBSs for CSO in the region, investing in other NBS plants and preparing a new regional regulation that invites to favor NBSs instead of grey solutions for CSO pollution control [13]; therefore, the Gorla Maggiore Water Park is an example of how a clear ES valuation could help to promote a real implementation of NBSs.

The message towards decision makers could be even more effective if the ES valuation were coupled with ES monetization, which can be done following a value transfer (VT) approach. According to the definition of Rolfe et al. [14], VT applies the quantitative estimates of an ES from an existing study (also called “study site”) to the site of interest (also called “policy site”). Examples of VT for CWs are the works of Ghermandi et al. [8], Ghermandi et al. [15], Woodward and Wui [16], Brander et al. [17], or He et al. [18]. Different VT methods are available, from the simplest unit value transfer to the more complex meta-analytic function transfer [19], with an overall quality of the results that may vary according to the objective of the VT [20,21]. Despite the higher quality of the results, complex VT methods, more suitable for critical steps in the decision-making context, such as accounting, priority-setting, instrument design, or litigation [21], require higher costs and time to be implemented. Therefore, simplified methods are suggested when the decision-making process takes place at an early stage. For instance, adjusted unit VT seems to be a valuable tool for a preliminary awareness raising. In order to investigate the potential role of adjusted unit VT as a tool to raise awareness on ESs and support decision making of CWs as multipurpose NBSs, this study reports the results from a feasibility study in which a recent ES valuation tool, B£ST [22,23], was used to guide the decision in choosing between green and grey infrastructure in an Italian case study.

## 2. Materials and Methods

### 2.1. Case Study

The feasibility study here presented was an action of Metro Adapt (www.lifemetroadapt.eu), a project funded by the LIFE Climate Change Adaptation Programme (2018–2021) and involving the Metropolitan City of Milan in partnership with the Water Utility (CAP Holding S.p.A.), associations (ALDA, Legambiente), and professionals (Ambiente Italia SRL, e-GEOS). A small-medium private enterprise expert in NBS, IRIDRA SRL, was subcontracted to investigate, with a feasibility study, the best option to treat a combined sewer overflow (CSO) in the municipality of Buccinasco, a small town in the Metropolitan area of Milan. The CSO was chosen after an interview with the Major of the Buccinasco Municipality, who highlighted the need to properly manage the CSO due to its critical location. Indeed, the CSO (45°25′5.61″ N, 9°7′43.3″ E) has a significant size, serving almost half of the inhabitants (about 27,000 in total), and is sited in the proximity of a residential area, leading to residents complaining. The CSO discharges mixed wastewater (i.e., both stormwater and domestic wastewater) generated by a sewer serving a total area of 73.1 hectares and a population equivalent (PE) of 11988. The industrial input is negligible, with a small part of industrial wastewater (less than 5% in terms of PE). 

### 2.2. Multi-Criteria Analysis (MCA)

The multi-criteria (or multi-attribute) analysis (MCA) involves the use of different types of variables aimed at providing a framework that allows to quantify preferences. This is particularly useful in the field of sustainability, where variables with different units are involved. Therefore, a MCA was used in this study to evaluate the most preferred alternative for the management of the CSO of Buccinasco. The literature framework of the MCA is discussed in the following sub-sections, in particular with regards to the use of value functions for the standardization of different units.

#### 2.2.1. Criteria Selection

Criteria were defined to compare different alternatives for the management of the Buccinasco CSO (Table 1). The selected criteria permitted to cover the important elements for the decision, including the main objective of the intervention (water quality), the NBS side benefits (air quality, biodiversity, carbon reduction and sequestration, education health, recreation, and wastewater treatment), and the negative impacts (CAPEX, OPEX and administrative issues).

Despite commonly used in MCA for CWs, the criteria for nuisance was neglected, since the proposed intervention is aimed at improving an already present nuisance condition, i.e., odor releases from the CSO discharge point. The areal footprint was also neglected, as it is already accounted for in the CAPEX criteria and is in line with EU policy of minimizing land consumption; indeed, the transformation of a non-natural area (urban or agricultural) into an NBS should be considered as an advantage, i.e., an occasion to provide ESs, rather than as a disadvantage as obstacle to future different anthropogenic uses of the land. For the same reasons, tax revenues were not considered as a criterion. Indeed, the alternatives were all located in areas not foreseen for urban development by the Municipality’s plans. Therefore, none of the alternatives were compromising future tax revenues from the land used.

#### 2.2.2. Alternative Definition and Sizing

Two groups of alternatives were defined, Alternative 1 for grey infrastructure and Alternative 2 for green infrastructure. Grey and green infrastructure are intended according to the definition given by Natural Water Retention Measures (nwrm.eu): grey infrastructure, solutions that use traditional methods to manage water, preventing any type of ecosystem from growing on it, and often built in concrete; green infrastructure, solutions that recreate natural or semi-natural areas to provide multiple services. Three alternatives were chosen for each group. The alternatives were sized in agreement with the recent Regional Regulations (R.R. 06/2019) as follows: the first flush tanks were sized on the basis of the impervious catchment, i.e., 29.3 hectares; in-line treatments (both green and grey infrastructures) were sized considering to continuously treat a CSO flow rate of 105 l/s, i.e., up to a dilution rate of 6 in comparison to the wastewater flow rate in the sewer during dry periods.

Grey infrastructure included both inline treatment and first flush tanks. As inline treatment, the option of primary treatment only (Alternative 1.1) was chosen, in order to consider the cheapest alternative but with the lowest water quality performance. Therefore, Alternative 1.1 included a preliminary automatic grid followed by a sedimentation tank of 375 m^3^, with an aerial footprint of 430 m^2^ (minimum hydraulic retention time: 1 h, targeted removal efficiencies: 50% TSS and 25% BOD_5_). Two first flush tanks were considered, following the minimum and maximum sizes indicated by the R.R. 06/2019, i.e., 25 and 50 m^3^ per impervious hectare. Consequently, Alternative 1.2 and Alternative 1.3 assumed a first flush tank of 730 m^3^ (areal footprint 540 m^2^) and 1460 m^3^ (areal footprint 880 m^2^), respectively.

Green infrastructures were chosen and sized following some of the most successful state-of-the art approaches used for CSO treatment with CWs [13]. Alternative 2.1 adopted the French approach, i.e., a single stage vertical subsurface flow constructed wetland (VF) with a net area of 3600 m^2^ and a total gross areal footprint of 7300 m^2^. Alternative 2.2 considered the Italian approach, i.e., a hybrid CW with a VF CW as 1st stage and a free water surface (FWS) system as 2nd stage; the net surfaces of the VF and the FWS were sized equal to 3600 m^2^ and 1500 m^2^, respectively, requiring a total gross areal footprint of 9550 m^2^. Alternative 2.3 also considered the Italian approach, but it was designed in a new park, following the example of the Gorla Maggiore Water Park [10,11]; the sizes of the VF and the FWS were the same as in Alternative 2.2, while the required total gross areal footprint was estimated equal to 19,750 m^2^. All the green infrastructures were assumed to provide the same high CSO treatment performance (TSS > 90%, BOD_5_ 50–70%), in line with recent state-of-the-art evidence [13].

**Figure 1:**
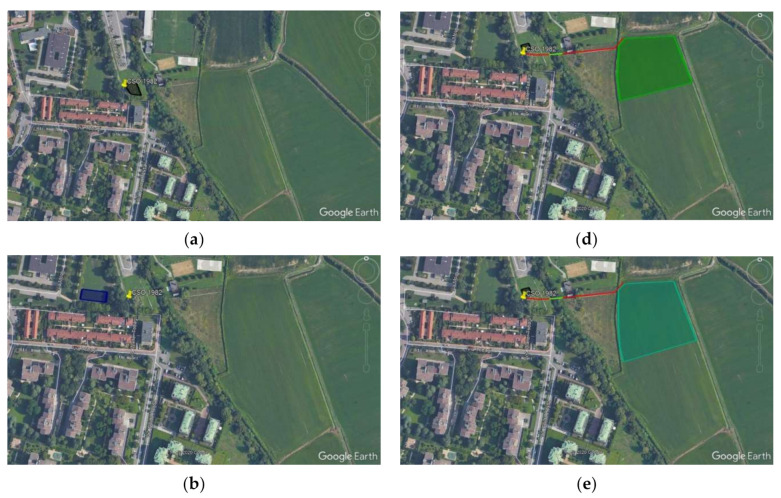

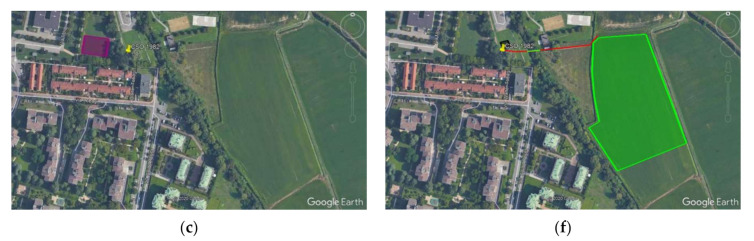
Plan view of the areal footprint and positioning of the different alternatives: (**a**) Alternative 1. 1, primary treatment only (primary only); (**b**) Alternative 1.2, first flush tank, minimum size according to local regulation (FFT—min); (**c**) Alternative 1.3, first flush tank, maximum size according to local regulation (FFT—max); (**d**) Alternative 2.1, constructed wetland with a single stage (VF); (**e**) Alternative 2.2, multistage constructed wetland (VF+ FWS); (**f**) Alternative 2.3, multistage constructed wetland sited in a newly developed urban park (VF + FWS park).

The plan views of the areal footprints are visible in Figure 1 for all the alternatives.

#### 2.2.3. Weights and Sensitivity Analysis

Five sets of weights were defined to perform a sensitivity analysis, which are shown in Table 1. For the first group, the weights were chosen to be representative of the case study with an expert-based approach (W1), i.e., considering the interview with the Major of the town of Buccinasco, a site visit, and the study of local context in terms of all the aspects of interest, such as environmental conditions, urban planning, and local legislation. The weights of the second group were assumed to be equally distributed, to investigate the best alternative in case of no preferences among the criteria. Finally, three sets of weights were defined to simulate the preferences of some particular stakeholders: (i) citizens (W3), more interested in social and welfare aspects; (ii) environmentalists (W4), who were assumed to prefer criteria related to environmental impact such as water or air quality; (iii) grey infrastructure professionals (W5), a general stakeholder group that was selected to highlight the “design as usual” preference, i.e., only considering costs in relation to the single main benefit of water quality.

### 2.3. Ecosystem Service Monetizaion

B£ST (Benefits Estimation Tool) is an open-access software developed by CIRIA (www.ciria.org), an English not-for-profit organization. B£ST allows to monetize up to 14 green-blue infrastructure ecosystem services and was already used for several applications by both professionals and researchers [22,23]. This study used the 5.1.1 version of the software, which was released in 2019. B£ST provides literature-based ESs values (study sites) to be transferred and suggests two correction factors (called confidence scores, with values to be set by users equal to 25%, 50%, 75%, or 100%) to fit the ES value to the local policy site. The first correction factor considers the confidence with the quantification of the ES (e.g., the number of people visiting a park) and the second takes into account the confidence with the monetized value to be transferred (how confident am I with the value of the study site to be transferred to the policy site?). According to the classification of VT methods given by Barton [24], B£ST monetizes the ESs with an approach that can be defined an “adjusted unit value transfer”, which transfers the values with simple adjustments and allows to consider the difference between policy and study sites with a simplified approach. If the confidence scores are not applied, B£ST simply transfers the value without any adjustments and can be classified as “unit value transfer”, i.e., when the values are transferred without any form of adjustment. 

The main assumptions used for ESs monetization are reported in Table 2. The central estimate of monetary values for VT was always chosen when B£ST provided a range (low, central, high). The reader is invited to consult the open-access B£ST Guidance for more details on the VT methodology and the transfer values for each ES. Transferred values of the ESs were converted from Pound to Euro considering an exchange rate of 0.85 £ €^−1^ (4 December 2019).

### 2.4. Other Criteria Evaluation

Investment costs (or CAPEX) were calculated detailing each cost item, based on parametric costs from regional price lists and IRIDRA’s experience in designing NBS for CSO treatment. Some furnishings for the park (alternative A2.3) were defined and priced. Finally, for the estimation of land acquisition, a reasonable land price was assumed for the local context based on the value given in other projects in the nearby areas, equal to 20 € m^−2^. Operational and maintenance costs (OPEX) were calculated following the approach used by Rizzo et al. [27], i.e., defining and pricing the expected O&M. Details of CAPEX and OPEX are reported for all the alternatives in Table 3.

Due to the larger area required, alternatives with green infrastructures needed to be placed where enough area was available, i.e., outside the Buccinasco borders and entering the Municipality of Milan. Therefore, an additional criterion was added, concerning potential administrative issues related to the implementation of an infrastructure outside the Buccinasco territory. The criterion was evaluated with a binary indicator and with a positive orientation: 0 (low) or 1 (high) if the alternative was outside or inside the Buccinasco borders. 

### 2.5. Value Functions and MCA Final Score

Value functions [28] were used to measure the preferences for each criterion among the different alternatives, i.e., to transform the criteria evaluation (effect matrix) into a degree of satisfaction (evaluation matrix). According to the methodology defined by Alacron et al. [29], value functions were built defining: (i) the orientation of the preference for each criterion (see Table 1); (ii) the relative points corresponding to the minimum (value 0) and maximum (value 1) performance/satisfaction among the alternatives; (iii) a linear shape.

Once each criterion was converted to dimensionless (0 lower preference, 1 higher preference), an MCA score for each alternative was calculated as follows

(1) $$\mathrm{MCA}\;\mathrm{Score}=\sum w_{i}v_{i}$$

where $w_{i}$ is the weight of the i-th criterion set in Table 1 and $v_{i}$ is the evaluation of the i-th criterion with the value function. Therefore, the MCA score can vary from 0 to 1, i.e., the minimum and maximum preference, respectively.

## 3. Results

### 3.1. MCA Results

**Figure 2:**
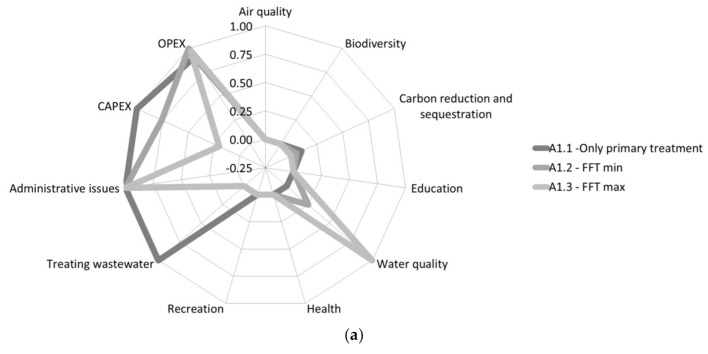

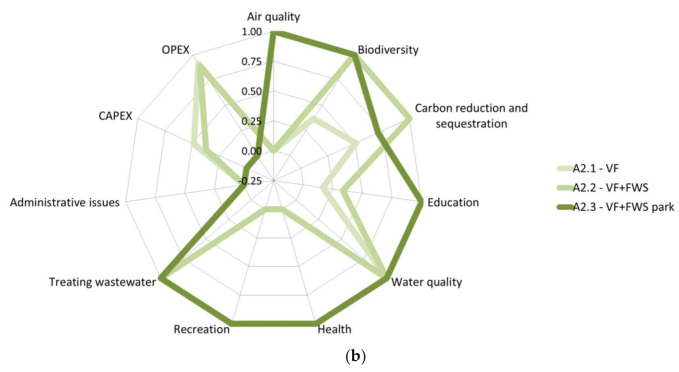
Graphical representation for the evaluation matrix of the MCA (after criteria normalizan. for the two groups of alternatives: (**a**) Grey infrastructure; A1.1: primary treatment only (primary only). A1.2: first flush tank, minimum size (FFT—min). A1.3: first flush tank, maximum (FFT—max). (**b**) Green infrastructure. A2.1: vertical subsurface flow CW (VF). A2.2, vertical subsurface flow plus free water surface CW (VF + FWS). A2.3, vertical subsurface flow plus free water surface CW in an urban park (VF + FWS park).

The effect matrix is reported in Table 4, including the ecosystem service monetization. The evaluation matrix, i.e., the evaluation of the criteria after the application of the value function, is graphically represented in Figure 2. The most interesting results and considerations are reported below:

- An improvement of air quality is expected only by A2.3, i.e., the only one for which the plantation of new trees was planned.
- Biodiversity support is given by all the alternative using green infrastructures. A2.1 is evaluated with a lower biodiversity contribution, as only one habitat (*Phragmites australis*) is implemented, with no free water wetland. Alternatives A2.2 and A2.3, instead, are better rated, since they also use a FWS stage, which is able to support a greater number of species (especially dragonflies).
- The best balance between CO_2_ production (due to energy consumption) and sequestration (biomass stock) results for the alternative A2.2, even if a positive balance is estimated for all the alternatives that use green infrastructure.
- Environmental education benefits result only for green infrastructures, due to the minimal ability to attract educational activities with grey solutions. The capability to propose environmental education activities increase with the increase of habitat complexity and recreational value of the area, i.e., from A2.1 to A2.3.
- All the alternatives give a positive contribution in terms of water quality. The lower effect of A1.1 is due to the lower performance of primary treatment only compared to the others.
- Health and recreation ESs are activated only by A2.3, i.e., only when the alternative includes an urban park and, therefore, is properly designed to provide these additional side-benefits.
- Benefits in terms of reduction of wastewater to be treated by the centralized WWTP are delivered only by the alternatives that provide a continuous in-line treatment, i.e., A1.1 and green infrastructures.
- Better performance of grey infrastructure are linked to criteria requiring a lower areal footprint, since the CAPEX are lower and there is no need to reach an agreement with the Municipality of Milan for the land located outside the Buccinasco Municipality border.
- OPEX range from the lowest value obtained for A1.2 (and slightly higher A1.3), and the highest obtained for the “urban park” alternative, which show O&M costs an order of magnitude higher. The other 3 in-line alternatives show similar performances.

**Figure 3:**
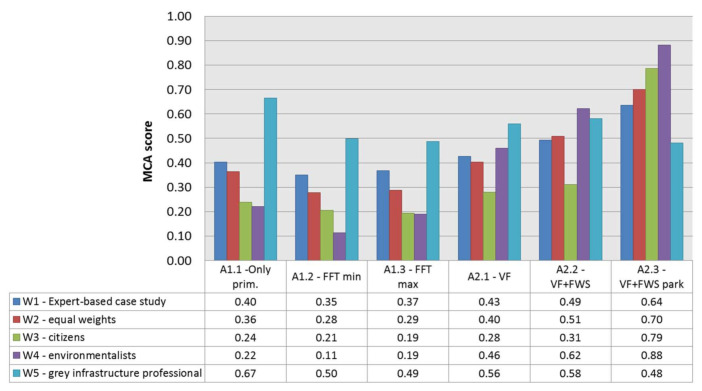
Results of the sensitivity analysis, with the MCA scores obtained assuming different weights for all the alternatives. A1.1: primary treatment only (only primary). A1.2: first flush tank, minimum size (FFT—min). A1.3: first flush tank, maximum (FFT—max). A2.1: vertical subsurface flow CW (VF). A2.2, vertical subsurface flow plus free water surface CW (VF + FWS). A2.3, vertical subsurface flow plus free water surface CW in an urban park (VF + FWS park).

Figure 2 shows the performance of each criterion, i.e., it is a graphical representation of the evaluation matrix for each alternative. Applying the weights chosen by experts (W1 of Table 1), the final MCA scores are, from highest to lowest (Figure 3): Alternative 2.3, 0.64; Alternative 2.2, 0.49; Alternative 2.1, 0.43; Alternative 1.1, 0.40; Alternative 1.3, 0.37; Alternative 1.2, 0.35. Hence, the creation of a green infrastructure in a new urban park is the best option for the management of the CSO of the Buccinasco town. 

Figure 2 clearly shows that grey and green infrastructures are preferable for different groups of criteria. Grey alternatives are comparable to green ones, or even better performing, in terms of cost-benefits, taking into account only the main-benefit, i.e., water quality, and neglecting all the side benefits. The multi-criteria approach here proposed allows highlighting additional ESs provided by green infrastructures, which should be accounted for to be properly compared with grey solutions. However, some ESs can be provided only when the design (and the investment) includes other citizens needs beside the CSO outflow treatment, taking the chance of building a treatment facility to create an urban park (Alternative 2.3). 

### 3.2. Sensitivity Analysis

The results of the sensitivity analysis are reported in Figure 3, where the MCA score is calculated for the set of weights defined in Table 1. Alternative 2.3 is largely dominating and remains the preferred one with equal weights (W2) as well as for citizens and environmentalist stakeholders (W3 and W4). Grey infrastructure can only be the preferred option by giving the 5 criteria where grey alternatives perform better a much greater relative importance than the others (“grey infrastructure professionals”, W5). Even using these weights, however, the preferred grey alternative is A1.1 (primary treatment only): that is the less effective solution for the main benefit (reduce pollution from the CSO). The second-best alternative is again a “green” alternative (A2.1, ranking above A1.2 and A1.3). However, the gaps of grey infrastructures (A1.1, A1.2, A1.3) compared to green ones not included in a park (A2.1, A2.2) are not so pronounced (almost always less than 0.2), highlighting the potential role of additional ESs provided by Alternative 2.3.

## 4. Discussion

The work had proposed a clear conceptual framework to support the decision on the possibility of using a NBS to solve a practical issue, i.e., the control of the pollution from the CSO of the Buccinasco town. In this way, the work is in line with the recent literature that is trying to clarify added values and limits of NBSs. According to the current needs posed to understand NBS potential role in social and environmental challenges, recently reviewed by Seddon et al. [30], the methodology tries to give a clear quantification of NBS effectiveness and cost-effectiveness, including those ESs which can be more difficult to be quantified, such as social services and biodiversity, and allowing a transparent comparison between green and grey solutions. The methodology was based on a Multi-Criteria Analysis, as done in Liquete et al. [11], and used a simplified value transfer (VT) approach to quantify and monetize ESs. According to Liquete et al. [11], green infrastructures are generally preferable to grey solutions. However, this study explores a broader set of alternatives, also including less multipurpose green infrastructure, i.e., more focused on the main target of water pollution control and less focused on possible secondary benefits. Although green infrastructures remain more desirable for most of the potential stakeholders (whose preferences have been simulated by different relative importance weights), the difference between green and grey infrastructure focusing only on wastewater treatment is rather small. On the other hand, taking the chance of building a treatment infrastructure to implement a multipurpose area provides other valuable social benefits (i.e., when the green infrastructure is part of a new urban park), in line with the higher willingness to pay registered by Reynaud et al. [12] when green infrastructures are included in park areas. The choice would move towards the simplest grey solutions (Alternative 1.1, primary treatment only, i.e., low cost and low water quality performance), if the preferences were completely mono-objective. Therefore, the results also help in going beyond the pitching of green solutions against grey ones, as recently suggested by Seddon et al. [30]. Green infrastructure is not better than the grey one per se, it simply provides multiple benefits for which, though, a site-specific interest should be verified. The presented results concern the use of land not planned for urban development, neglecting the role of tax revenues in the decision making process. If either green or grey infrastructure solutions involved an urban development area, the loss of tax revenues would be considered as an additional criterion of the MCA analysis. On the other hands, CSOs and receiving streams should be planned in green (infrastructure) areas sufficiently far from buildings, therefore, the issue of tax revenue should be less relevant for properly planned future sustainable cities. 

VT is a powerful tool to quantify and monetize ESs, helping to have a transparent comparison between green and grey solutions. Complex methodologies, such as meta-analytic analysis [8,15,16,17], seem not suitable for an early stage rise of the multipurpose NBS potentialities. To this aim, simplified methods (adjusted unit VT) and software (e.g., B£ST) seem more appropriate due to required lower costs and efforts. Therefore, adjusted unit VT can be viewed as a simplified method to raise awareness on additional ESs that can be designed with a NBS from the early stages of the decision, such as the feasibility study of Buccinasco here proposed. At the same time, simplified VT methods should always be used with caution, and it is suggested to consult experts of ES evaluation for this activity. Table 5 shows the results of the ESs monetization with and without the use of the expert-based confidence scores, i.e., adjusted unit VT and simple unit VT. The results of the adjusted unit VT are realistic, particularly in terms of cultural ESs, which are estimated variable from 149 € y^−1^ ha^−1^ to 6984 € y^−1^ ha^−1^ (Alternative 2.1 to 2.3, respectively), i.e., in line with the values reviewed by Ghermandi et al. [8] for CWs (median 530 € y^−1^ ha^−1^, mean 8397 € y^−1^ ha^−1^). Interestingly, the results obtained with the adjusted unit VT suggest that green infrastructures are capable of creating value from the ESs of the same order of magnitude as the OPEX. Contrarily, if the simpler and optimistic unit VT had been used, the ES monetization would probably have been overestimated, especially for more uncertain cultural ESs (education, health, recreation). In this case, the risk would be to create too high and unrealistic expectations from the NBS.

Table 5 also suggests that care should be given in presenting the results from a VT and in using VT as a tool to quantify criteria in MCA. The methodology is, *per se*, a transfer of the interest of human-being in ESs. As a consequence, cultural ESs generate higher monetization then other ES groups. This should not lead to the mistake of considering other ESs negligible; for instance, the value of the biodiversity support from NBS can be high from an environmental perspective, independently from the value sensed (i.e., monetized) by citizens. Therefore, ES monetization should always be used, in MCA, with a relative normalization in the value function definition, not mixing the maximum and minimum monetized values across the different ESs.

## 5. Conclusions

A Multi-criteria analysis (MCA) was performed to compare grey and green infrastructure alternatives for the management of a combined sewer overflow in the Buccinasco town (Italy), in which the criteria related to ESs were monetized with an adjusted VT method (B£ST software). The results highlighted the potential interest in the implementation of the green infrastructure in a new urban park, due to the activation of additional ESs of interest, such as health and recreational aspects.

Without a MCA and a simple tool as B£ST, the decision maker would probably not even consider the possibility to create a new park for the citizens of Buccinasco, exploiting the possibility of implementing a new NBS. The water utility (CAP Holding) is now discussing with the Buccinasco Municipality about the implementation of the best ranked alternative, i.e., an NBS in a new urban park. Moreover, CAP Holding is also using the results of this feasibility study to support the decision to implement an NBS for a CSO in a new park in a nearby town, the Paderno Dugnano Municipality.

## Figures and Tables

**Table 1:** Criteria (with orientation and indicators) and weights defined for the sensitivity analysis, expressed in absolute (A: scale 0–10) and normalized (N: scale 0–1) terms.

Criteria	Orient.	Indicator	W1		W2		W3		W4		W5	
			A	N	A	N	A	N	A	N	A	N
Air quality	+	€ life span	7	0.09	10	0.09	10	0.18	10	0.13	2	0.03
Biodiversity	+	€ life span	5	0.06	10	0.09	2	0.04	10	0.13	2	0.03
C reduction and sequestration	+	€ life span	5	0.06	10	0.09	0	0.00	10	0.13	2	0.03
Education	+	€ life span	7	0.09	10	0.09	7	0.13	7	0.09	2	0.03
Water quality	+	€ life span	10	0.12	10	0.09	3	0.05	10	0.13	7	0.12
Health	+	€ life span	7	0.09	10	0.09	10	0.18	7	0.09	2	0.03
Recreation	+	€ life span	7	0.09	10	0.09	10	0.18	5	0.07	2	0.03
Wastewater treatment	+	€ life span	5	0.06	10	0.09	2	0.04	10	0.13	10	0.17
Administrative issues	-	Expert judgment	8	0.10	10	0.09	0	0.00	0	0.00	10	0.17
CAPEX	-	€	10	0.12	10	0.09	5	0.09	2	0.03	10	0.17
OPEX		€ y^−1^	10	0.12	10	0.09	7	0.13	4	0.05	10	0.17

W1 Expert-based (Case study). W2 Equal weights. W3 Citizens. W4 Environmentalists. W5 Grey infrastructure professionals. +: positive orientation, i.e., higher value of the indicator, higher preference of the criterion. -: negative orientation, i.e., lower value of the indicator, higher preference of the criterion.

**Table 2:** Assumptions used in B£ST for the value transfer (VT) of ecosystem services (ESs) for the different MCA alternatives, including quantity (Q) and valuation in £ (V) confidence scores. A1.1: primary treatment only (primary only). A1.2: first flush tank, minimum size (FFT—min). A1.3: first flush tank, maximum (FFT—max). A2.1: vertical subsurface flow CW (VF). A2.2, vertical subsurface flow plus free water surface CW (VF + FWS). A2.3, vertical subsurface flow plus free water surface CW in an urban park (VF + FWS park).

ES	Confidence Scores ^1^		Assumed Information for VT	
	Q	V	Type/Proxy	Value
Air quality	50%	100%	no. and type of trees	A2.3 no. 100 trees tree type, medium No effect for all other alternatives
Biodiversity	50%	25% VF 100% FWS	wetland area	A2.1, A2.2, A2.3 From low (arable fields) to high (wet reed beds) No effect for all other alternatives
Carbon reductionand sequestration	25% trees 50% CW	100%	no. and type of trees energy consumption wetland area	All the alternatives CO_2_ production ^2^ and reduction due to energy consumption ^3^ A2.1, A2.2, A2.3 C stock of CW biomass ^4^ A2.3 no. 100 trees tree type, medium
Education	25%	50%	no. of studentsper year	Environmental education activities of the group of interest ^5^: A2.1, no. 110 students per year (no.1 visit per year for each group of interest) A2.2, no. 210 students per year (no.2 visit per year for each group of interest) A2.3, no. 660 students per year (no.6 visit per year for each group of interest) No visits for grey infrastructure alternatives.
Water quality	25%	25%	no. of NWEBS categories with a change in water quality classification ^6^ Local context Length of the water course with improved quality	Change in water quality classification: Bad to Poor no. of NWBES changes: A1.1: no.1 (clarity of the water) A1.3, A2.1, A2.2, A2.3: no.3 (clarity of the water, invertebrates, plant communities) A1.2: no.2 (proxy value to consider the expected lower effect on water quality of the smaller first flush tank in comparison to A1.3) Local context: South East of UK ^7^ 0.5 km of water course with improved quality ^8^
Health	25%	50%	no. of visits per year	A2.3 ^9^ no. 3398 visits per year for physical activity no. 3398 visits per year for emotional wellbeing No effect for all other alternatives
Recreation	25%	50%	no. of visits per year	A2.3 ^9^ no. 4757 visits per year to urban green space no. 2039 visits per year to freshwater No effect for all other alternatives
Treating wastewater	50%	100%	Daily average flow not discharged in sewer	A1.2, A2.1, A2.2., A2.3 –287 m^3^ d^−1^ not discharged in sewer ^10^ No effect for the alternatives using a first flush tank (A1.2, A1.3)

^1^ Selected according to the guidelines given in the B£ST Guidance. ^2^ Preliminary grit: 4 kW, 1200 kWh y^−1^ (A1.1, A2.1, A2.2, and A2.3). Pumps for emptying the first flush tank: 2 kW, 3360 kWh y^−1^ (A1.2 and A1.3). Pumps for VF CW feeding: 7.5 kW, 2100 kWh y^−1^ (A2.1, A2.2, A2.3). Lighting of the park with LED: 0.15 kW, 16,425 kWh y^−1^ (A2.3). ^3^ B£ST estimates of the reduction in energy consumption within the treating wastewater benefit evaluation. ^4^ Net C sequestration of CW equal to 1 kg_C_ m^2^ y^−1^ [25]. ^5^ The groups potentially interested in environmental education activities were defined according to the census of local schools and associations: no. 2 elementary schools (no. 15 students per visit); no. 2 middle schools (no. 15 students per visit); no. 1 scout association (no. 50 scouts per visit). ^6^ Categories of the NWEBS (National Water Environment Benefits Survey): (i) fish; (ii) invertebrates; (iii) plant communities; (iv) clarity of the water; (v) river channel conditions and water flow; (vi) water safety for recreational contact. ^7^ B£ST targets UK. Therefore, the South-East region was selected as a *proxy* for the Buccinasco study, as the presence of London in this region can be compared to the presence of Milan near the town of Buccinasco. ^8^ That is the length from the nearest CSO downstream. ^9^ The potential visits to the new planned urban park were estimated defining a potential number of visits per year with the following assumptions: users potentially interested in visiting the park equal to 50% of the inhabitants of the nearby neighborhood, considering an accessibility distance of 1 km [26], i.e., equal to 1132 inhabitants; no.1 visit per month for each potential user, i.e., no. 13,590 visits per year; 25% of visits per year for physical activity (health), 25% for emotional wellbeing (health), and 50% for recreation; among the yearly recreational visits, 70% for urban green space, and 30% for freshwater. ^10^ Treated and discharged CSO volume equal to 14,895 m^3^ y^−1^ treated in-line, which would be intercepted and discharged into the sewer network if the first flush tanks were used.

**Table 3:** Detailed CAPEX and OPEX estimation for all alternatives. A1.1: primary treatment only (prim. only). A1.2: first flush tank, minimum size (FFT—min). A1.3: first flush tank, maximum (FFT—max). A2.1: vertical subsurface flow CW (VF). A2.2, vertical subsurface flow plus free water surface CW (VF + FWS). A2.3, vertical subsurface flow plus free water surface CW in an urban park (VF + FWS park).

Item	A1.1 prim. Only	A1.2 FFT min	A1.3 FFT max	A2.1 VF	A2.2 VF + FWS	A2.3 VF + FWS Park
Grid	100,000 €	- €	- €	100,000 €	100,000 €	100,000 €
Sedimentation tank	190,000 €	- €	- €	- €	- €	- €
Grit chamber	- €	- €	- €	25,000 €	25,000 €	25,000 €
First flush tank	- €	512,000 €	1,024,000 €	- €	- €	- €
Pumping station	- €	- €	- €	30,000 €	30,000 €	30,000 €
Pumps	- €	- €	- €	60,000 €	60,000 €	60,000 €
Piping	- €	- €	- €	44,000 €	44,000 €	44,000 €
Culvert	- €	- €	- €	10,000 €	10,000 €	10,000 €
VF	- €	- €	- €	360,000 €	360,000 €	360,000 €
FWS	- €	- €	- €	- €	60,000 €	60,000 €
Pedestrian path	- €	- €	- €	- €	- €	10,000 €
Bike trail	- €	- €	- €	- €	- €	15,000 €
Rest area	- €	- €	- €	- €	- €	6000 €
Trees	- €	- €	- €	- €	- €	25,000 €
Benches	- €	- €	- €	- €	- €	3000 €
Racks	- €	- €	- €	- €	- €	500 €
Lighting	- €	- €	- €	- €	- €	13,000 €
Playground	- €	- €	- €	- €	- €	20,000 €
Boardwalk	- €	- €	- €	- €	- €	50,000 €
Land acquisition	8500 €	8800 €	17,600 €	146,000 €	191,000 €	395,000 €
Total CAPEX	298,500 €	520,800 €	1,041,600 €	775,000 €	880,000 €	1,226,500 €
Energy ^1^	200 € y^−1^	500 € y^−1^	900 € y^−1^	600€ y^−1^	600 € y^−1^	3900 € y^−1^
Sludge removal ^2^	1500 € y^−1^	- € y^−1^	- € y^−1^	- € y^−1^	- € y^−1^	- € y^−1^
Reed harvesting ^3^	- € y^−1^	- € y^−1^	- € y^−1^	1100 € y^−1^	1100 € y^−1^	1100 € y^−1^
Green maintenance ^3^	- € y^−1^	- € y^−1^	- € y^−1^	100 € y^−1^	200 € y^−1^	7500 € y^−1^
Personnel ^4^	900 € y^−1^	900 € y^−1^	900 € y^−1^	700 € y^−1^	900 € y^−1^	1500 € y^−1^
Total OPEX	2600 € y^−1^	1400 € y^−1^	1800 € y^−1^	2500 € y^−1^	2800 € y^−1^	14,000 € y^−1^

^1^ Preliminary grit: 4 kW, 1200 kWh y^−1^ (A1.1, A2.1, A2.2, and A2.3). Pumps for emptying the first flush tank: 2 kW, 3360 kWh y^−1^ (A1.2 and A1.3). Pumps for VF CW feeding: 7.5 kW, 2100 kWh y^−1^ (A2.1, A2.2, A2.3). Park lighting with LED: 0.15 kW, 16,425 kWh y^−1^ (A2.3). Energy cost: 0.2 € kWh ^−1^. ^2^ It is assumed that 20% percent of the sedimentation volume is removed as sludge each year. Parametric cost of sludge removal, transport and unload: 20 € m^−3^. ^3^ CW and green harvested biomass: 3 and 2 kg m^−2^, respectively. Parametric cost for reed and green harvest: 0.1 € m^−2^. Parametric cost for transport, load and unload: 18 € ton^−1^. Parametric cost for waste in landfill: 50 € ton^−1^. Width of mowed area around CW: 2 m. ^4^ Time per visit: 4 h of non-specialized personnel (25 € h^−1^). Number of visits after heavy rain events: 3 per year for all the alternatives. Number of ordinary visits: A1.1, A1.2, A1.3, A2.2, every 2 months; A2.1, every three months; A2.3, every month.

**Table 4:** Effect matrix of MCA, including results of value transfer for ecosystem services, for all the alternatives.

Criteria	Orient.	A1.1 prim. Only	A1.2 FFT min	A1.3 FFT max	A2.1 VF	A2.2 VF + FWS	A2.3 VF + FWS Park
Air quality ^1^	+	0 €	0 €	0 €	0 €	0 €	11,240 €
Biodiversity ^1^	+	0 €	0 €	0 €	264 €	719 €	719 €
Carbon reduction and sequestration ^1^	+	- 84 €	- 209 €	- 334€	919 €	2139 €	1420 €
Education ^1^	+	0 €	0 €	0 €	5254 €	10,508 €	31,525 €
Water quality ^1^	+	1786 €	2679 €	5359 €	5359 €	5359 €	5359 €
Health ^1^	+	0 €	0 €	0 €	0 €	0 €	141,099 €
Recreation ^1^	+	0 €	0 €	0 €	0 €	0 €	73,085 €
Wastewater Treatment ^1^	+	19,021 €	0 €	0 €	19,021 €	19,021 €	19,021 €
Administrative issues	+	1	1	1	0	0	0
CAPEX	-	298,500 €	520,800 €	1,041,600 €	775,000 €	880,000 €	1,226,500 €
OPEX	-	2600 € y^−1^	1400 € y^−1^	1800 € y^−1^	2500 € y^−1^	2800 € y^−1^	14,000 € y^−1^

^1^ Ecosystem service monetization assuming 21 years as an evaluation time frame (from 2019 to 2040). A1.1: primary treatment only (primary only). A1.2: first flush tank, minimum size (FFT—min). A1.3: first flush tank, maximum (FFT—max). A2.1: vertical subsurface flow CW (VF). A2.2, vertical subsurface flow plus free water surface CW (VF + FWS). A2.3, vertical subsurface flow plus free water surface CW in an urban park (VF + FWS park). +: positive orientation, i.e., higher value of the indicator, higher preference of the criterion. -: negative orientation, i.e., lower value of the indicator, higher preference of the criterion.

**Table 5:** Ecosystem service (ES) monetization estimated with B£ST for green infrastructure alternatives, considering and not considering confidence scores, i.e., Unit value Table 2. 1: vertical subsurface flow CW (VF). A2.2, vertical subsurface flow plus free water surface CW (VF + FWS). A2.3, vertical subsurface flow plus free water surface CW in an urban park (VF + FWS park).

Alternative	VT Method	ES Prov. ^1^	ES Regul. ^2^	ES Cult. ^3^	ES Supp. ^4^	ES Total		OPEX
		[€ y^−1^]	[€ y^−1^]	[€ y^−1^]	[€ y^−1^]	[€ y^−1^]	[€ y^−1^ ha^−1^] ^5^	[€ y^−1^]
A2.1 VF	U	2131	4933	2355	61	9480	12,986	2552
	AU	1066	352	294	15	1726	2365	
A2.2 VF + FWS	U	2131	4933	4710	86	11,860	12,419	2848
	AU	1066	412	589	40	2115	2214	
A2.3 VF + FWS park	U	2131	6442	110,350	86	119,010	60,258	13,955
	AU	1066	981	13,794	40	15,881	8041	

^1^ ES Provisioning: Asset performance (“Pumping” and “Treating wastewater”). ^2^ ES Regulating: “Air quality”, “Carbon reduction and sequestration”, and “Water quality”. ^3^ ES Cultural: “Education”, “Health”, “Recreation”. ^4^ ES Supporting: “Biodiversity and ecology”. ^5^ Calculated on the gross area of the NBS.

## Data Availability

Not applicable.

## Abbreviation

CSO	combined sewer overflow
CW	constructed wetland
ES	ecosystem services
MCA	Multi-criteria Analysis
NBS	Nature-based Solution
VT	value transfer
